# A Comprehensive Review of the Importance of Selected Trace Elements Present in Edible Insects

**DOI:** 10.1007/s12011-022-03423-z

**Published:** 2022-09-16

**Authors:** M. Mabelebele, S. D. Kolobe, E. Malematja, N. A. Sebola, T. G. Manyelo

**Affiliations:** Department of Agriculture and Animal Health, College of Agriculture and Environmental Sciences, University of South Africa, Florida, 1710 South Africa

**Keywords:** Insects, Edible insects, Minerals, Trace elements

## Abstract

This review is intended to provide recent published information on trace elements from edible insects from various environments. Recently, insects are gaining popularity as food proteins in developing countries and press higher demand for edible insects since they may provide similar nutritional value as meat. Insects have been part of the human diet in the world for decades and at least 1900 insect species are considered edible. Furthermore, insects play a crucial role in socioeconomic by contributing to the world’s food security as well as eradicating poverty in rural communities. Generally, edible insects are considered a readily available source of proteins, carbohydrates, and chitin. They also contain considerable amounts of trace elements such as iron, zinc, copper, and manganese. It has been observed that there is a great variation between mineral contents found in insects of the same or different species. Knowledge and comprehensive understanding of trace element contents of edible insects are crucial to fully maximise their utilisation in diets and prevent mineral deficiency in human beings and animals. However, most of the research on insects has focused on the nutritional contents of insects with less attention given to other nutritional components such as minerals and trace elements. The available data on trace elements from edible insects as food is limited and makes it difficult to draw estimations for the nutrient intake of humans and animals. Therefore, this review aimed to provide comprehensive information on availability of iron, zinc, copper, and manganese from selected edible insects, functions, and deficiencies in both humans and animals.

## Introduction

Edible insects have been part of the human diet of the world for decades and at least 1900 insect species are considered edible [1–3] and about 2 billion people across the world consume edible insects on regular basis [4]. Insects contribute toward global food security as well as eradicating poverty in rural communities by providing a source of income to traders and harvesters [5]. In another literature on edible insects, Nowak et al. [6] indicated that insects serve as protein food in many cultures worldwide and their consumption is increasing at an alarming rate. From the animal production perspective, insect meals are considered cheap, desirable, and acceptable to potentially be utilised as a protein source for livestock, especially poultry species [7]. They are mainly characterised by type species and metamorphosis stage and higher levels of trace elements than meat [3]. The most common insect orders include lepidopterans, orthopterans, isopterans, and hymenopterans [8]. Insects undergo a metamorphosis process with various growing stages before they reach the adult stage, and they are edible in all those different stages [6]. The pupae and larvae stage are mostly preferred for consumption as the nutritional content at this stage is high and produces a unique flavour and taste [8]. In South Africa, edible insects are more consumed in Limpopo province by vhaVenda, Bapedi, and Vatsonga people, and certain insect species are consumed by specific tribes, for instance, stinkbugs are being consumed mostly by vhaVenda [5]. Edible insect species consumed in South Africa include mopane worms (*Imbrasia* spp.), termites (*Macrotermes* spp.), grasshoppers (*Schistocerca gregaria*), and stinkbugs (*Encosternum delegorguei*) [9]. However, some societies perceive insect consumption as culturally inappropriate or as disgusting, especially in urban areas [4]. Despite the insect consumption being suppressed by some societies, insects are currently gaining popularity as food proteins in developing countries and press higher demand for edible insects’ the reason being a reduction of meat consumption because insects may provide similar nutritional value as meat [4, 10, 11]. It has been reported that insects have high feed conversion efficiency than livestock which makes them suitable for rearing on a large scale and their market is increasing [12].

In addition, insects are appreciated for their content of potential minerals meeting some nutrient requirements in humans and animals as well [11]. Edible insects and arachnids contain adequate minerals including trace elements that meet the dietary requirement of captive insectivores, pets, or poultry [13]. Minerals are divided into two classes based on the amount needed to meet dietary requirements. The first class is macro-minerals, namely calcium, phosphorus, magnesium, sodium, potassium, and chlorine which are measured in g per kg. The second class consists of micro or trace minerals, namely iron, zinc, copper, manganese, iodine, and selenium which are measured in mg/100 g or mg/kg [14]. Most insect species from different orders are rich in trace elements such as iron, zinc, copper, and manganese needed to meet mineral requirements by animals [4, 14–16]. These trace elements are essential for the proper functioning of the physiological systems occurring in the body including the hormonal system, neuromuscular system, and reproductive system [1, 17, 18]. It has been observed that insects could potentially provide a large quantity of minerals such as iron contents recommended for human consumption [19], since the body requires small quantities of trace elements to support proper growth and development, enzymes and hormones production, as well as other biological processes within the body [2, 18, 20]. Therefore, the main focus of this review is on the following trace elements: iron, zinc, copper, and manganese.

However, there is a greater variation and inconsistency in the number of trace elements found in insects of the same or different insect species [14, 21, 22]. This is caused by factors such as the species type, diets, genetic factors, metamorphosis stage, feeding habits, species gender, environmental conditions, geographical location, soil composition, vegetation type, and processing methods, which significantly influence their nutritional compositions [1, 5, 16, 18, 21, 22]. There is little information about the utilisation of insect species such as mopane worms (*Imbrasia* spp.), termites (*Macrotermes* spp.), grasshoppers (*Schistocerca gregaria*), and stinkbugs (*Encosternum delegorguei*) as a source of microminerals in various countries, especially in southern Africa [5, 14]. Although there is an increasing number of scientific research and enterprises in the production of insects and insect products for humans and animal food [23, 24], most of the research on feeder insects has focused on the nutritional contents of insects with less attention given to other nutritional components such as minerals and trace elements [13]. Data available on trace elements from edible insects as food is limited and makes it difficult to draw estimations for the nutrient intake of humans. Therefore, the present paper is intended to provide recently published information on iron, zinc, copper, and manganese present in different edible and feeder insects and their utilisation.

## Potential Insects as a Source of Trace Elements

Micronutrients (trace elements) are considered vital for the normal functioning of organism’s body [1, 18, 25]. Fe and Zn are considered the most vital trace elements worldwide due to the role they play in the body to maintain proper human health [26]. Mineral and trace element analysis of commercially reared edible and feeder insects are being taken into consideration due to their role as food or feed supplement for captive insectivores in zoos, pets, or poultry birds [27–29]. Nutritional analysis of commercially bred feeder insects indicates that most insects are an excellent source of minerals and trace elements [28]. Insects are mainly rich in protein and amino acids; however, they have considerable amounts of other nutrients such as minerals [30]. The high protein content in edible insects has been reported to be associated with improving the uptake of microminerals such as Fe, Mn, Cu, and Zn levels [18, 31]. This means that the amount of protein found in insects could also help make assumptions about their microminerals contents [2]. Furthermore, micronutrient concentrations in insects were found to be positively correlated, for example, Fe concentrations increase along with Zn levels [2, 14]. However, the information about the quantity of zinc compared to that of iron contents found in various insects is also limited [21]. Trace elements found in edible insects are readily available and can help replace microminerals lost in the body to maintain the balance of elements content storage in the body [18]. According to Mwangi et al. [2], Fe and Zn in insects are available in non-haemoglobin forms (similar to that in plants) attached to proteins, whereas the iron-type contained in meat products is in haem iron form [21].

Although beef meat was reported to provide more digestible Fe than poultry meat, edible insects such as mopane worm (*Gonimbrasia belina larvae*) have been observed to contain higher iron levels (51.05 mg/100 g) than meat from livestock [3]. Similar findings also reported that insects contain higher iron and calcium contents compared to beef, pork, chicken, and other expensive protein sources [5, 30, 32]. Moreover, the iron solubility rate of insects is much higher compared to beef [19]. However, it has been reported that there is a significant variation in trace element contents such as Fe levels between different insect species [18]. Although insects are rich in microminerals, there is limited or no information about the utilisation of insects as sources of trace elements in the human diet [2, 21]. Some of the common documented edible insects rich in trace elements include beetles, caterpillars, ants, grasshoppers, locusts, crickets, stinkbugs, termites, flies, and cockroaches [3, 5, 33, 34]. For example, edible insects such as termites, mopane worms, house crickets, yellow mealworms, and locusts contain extremely high Mn contents compared to other insects [14, 22, 35]. According to Payne et al. [22], high Mn in insects may be caused by factors such as soil and water composition found in the origin of insects. The following are some of the most commonly documented edible insect species and their various trace elements.

1. *Termites*
  Termite species fall under Isoptera (Termitidae) order. Most termites are found in abundance during the spring in rainy seasons and can be differentiated by their morphological features, time, and season of emergence [1]. The most common edible termite species includes *Macrotermes species*; *Macrotermes nigeriensis*, *Macrotermes notalensis*, *Macrotermes subhylinus*, and *Macrotermes bellicosus*, *Ruspolia nitidula*, and *Brachytrupes species* [1, 36]*. Macrotermes alates* and *Macrotermes soldiers* are some of the termite species already evaluated for their nutritional status [22]. It has been reported that *Macrotermes bellicosus* termite species are rich in trace element contents, especially iron concentration [5]. Another study observed that the Fe content found in termite *Macrotermes subhyalinus* is significantly high and varies with its Zn contents [2]. However, according to Rumpold and Schluter [30], only fewer termite insect species are rich in iron contents. Termites endemic to Africa have been reported to be rich in zinc concentrations [5, 37–40]. It has been observed that their various termites found in the same geographical region differ in trace element composition such as zinc and iron levels [5]. Moreover, *Nasutitermes* species are reported to contain low Mn concentration compared to other termite species. A study observed that Mn concentration in alate termites is less than that of termite workers [14]. Similarly, Rumpold and Schluter [30] reported that *Macrotermes nigeriensis* termites are reported to have low manganese contents with a sufficient amount of selenium in mg/100 g dry matter.

### Grasshopper/Locusts and Crickets

The grasshoppers, locusts, and crickets fall under the Orthoptera order [16]. It has been reported that insect meals from Orthoptera order contain high content of various essential nutrients including trace elements [7]. Insects from Orthoptera have higher iron that is readily available to maintain and balance iron in the body [18]. According to Rumpold and Schluter [30], they have high levels of Zn and better Mn and Cu contents. Furthermore, locust species including *Locusta migratoria* is rich in iron content compared to beef [18, 41]. Similar findings were reported by Mwangi et al*.* [2] who reported that locusts and crickets contain similar Zn and higher Fe contents compared to chicken, pork, and beef. In addition, locusts have been observed to contain lower Fe content as compared to other insects such as yellow mealworm [18]. According to Kim [42], *Oxya chinensis sinuosa* grasshoppers contain high amounts of Fe, Mn, and Mo. Sun et al. [43] also observed an increase in total iron content of broiler chicken meat-fed grasshoppers compared to chicken meat without grasshopper inclusion in diets. The inclusion further improved the meat shelf life of broilers [7]. It could be concluded that various locust species are rich in iron, zinc, copper, and manganese. They can provide a sufficient amount of trace elements compared to most edible insects.

2. *Silkworm*
  The silkworm is the larvae phase of an adult moth [8]. According to Elahi et al. [7], the larvae feed on mulberry leaves and have been reported to be rich in nutrients including microminerals. The most important developmental stage of this insect is the larvae named silkworm due to its ability to produce the silk used in the formation of cocoon [17]. Silkworm species vary greatly in mineral content including trace elements due to various factors such as geographical location, seasonality, and diet [44, 45]. The species at pupae levels have high manganese and zinc content with a considerable amount of iron required for human health and nutrition [46]. Trace elements and vitamin contents in commercially produced silkworms can be manipulated and controlled via feeding to improve their nutritional status [46]. This edible insect is mainly high in zinc levels, especially at the larvae stage. However, fortunately, other essential trace elements such as zinc and iron contents within its body can successfully be altered to improve its mineral utilisation by human or animal feeding.
3. *Other Edible Insects*

Other edible insects include worms from order Lepidoptera and bugs from Hemiptera which are known to contain considerable levels of minerals including trace elements, respectively [9, 22, 28, 47]. Kröncke et al. [11] investigated the mineral profile of yellow mealworm and stated that these insect species could be considered a source of Zn, Fe, Mn, and Cu. These data are consistent with the results made by Oonincx [31] who investigated the chemical composition of insects as alternative food and observed that insects contain a sufficient amount of trace elements such as Zn, Fe, Mn, and Cu for various domesticated birds. However, insects have insufficient calcium to phosphorus ratio which could lead to calcium deficiency without supplementations. On proximate analysis, *Tenebrio molitor* larvae have about 34.8% of minerals and trace elements [6]. Mopane worms (*Gonimbrasia belina*) are known to be rich in terms of protein and trace elements such as magnesium, zinc, and iron [47]. In most feeder insects, magnesium is the most abundant element and followed by zinc [27, 28]. Stinkbugs are also rich in trace elements with high zinc and iron contents which can be promoted as an essential part of the human diet [22]. According to Rumpold and Schluter [30], insects such as stinkbugs have high contents of magnesium. Various edible insects such as mealworms, mopane worms, crickets, and stinkbugs contain high levels of trace elements mainly zinc, iron, manganese, and copper. Which are the most essential microminerals required by the body to inhibit mineral deficiencies for better nutrition and health. Trace element analysis on edible insects from various literature is shown in Table 1.

**Table 1:** Trace elements composition of selected edible insect species on a dry basis (mg/100 g)

Insect species	Developmental stage	Composition				Authors
		Zn	Cu	Mn	Fe	
Grasshoppers						
* Zonocerus variegatus*	Adult	0.36–1.71	0.20	-	1.75–2.32	[48–50]
* Valanga nigricornis*	Nymph	-	-	-	3.20	[18]
* Ruspolia differens*	Adult	12.4–17.3	0.5	2.5	13–16.6	[2, 8, 30, 51]
* Sphenarium purpurascens*	Adult	42.00	-	-	18.00	[2]
* Oxya chinensis sinuosa (mg/kg)*	Adult	110.0	27.2	48.2	99.7	[42]
* Oxya hyla hyla*	Adult	17.34	4.36	2.30	16.19	52
* Sphenarium histrio*	Adult	78.00	-	-	16.00	[8]
Grasshoppers (mg/kg) (unspecified)	Adult	256.92	73.02	-	349.27	[33]
Locusts						
* Brachystola magna*	Nymph	9.30	3.80	-	44.64	[53]
* Nomadacris succincta*	Nymph	-	-	-	3.15	[18]
* Locusta migratoria*	Adult	14.8–25.0	6.0	1.0	9.2–13.7	[2, 35]
* Schistocerca gregaria*	Adult	3.68–18.6	-	3.57	4.83–8.38	[2, 54]
* Bombay Locust*	Adult	8.22	1.69	1.07	3.45	[44]
* Locu (mg/kg)*	Adult	1.60	9.90	-	5.75	[33]
Crickets						
* Acheta domesticus*	Nymph	14.95–30.2	0.88–6.91	2.5–4.59	1.8–51.9	[2, 8, 11, 13, 28, 33]
* Gryllus dssimilis*	Adult	5.22–15.57	0.68	1.42	2.78–17.93	[2, 55–57]
* Gryllus bimaculatus*	Adult	22.43	4.55	10.36	9.66	[55–57]
Termites						
* Brachytrupes spp.*	Adult	23.02	-	-	33.60	[1]
* Macrotermes* spp.	Adult	13.8–38	6–8.2	292.7–714	10.3–40	[22, 35]
* Macrotermes subhyalinus*	Adult	8.1–10.3	8.5	422	13.4–61.9	[2, 35, 51, 58]
* Macrotermes bellicosus*	Adult	10.76–16.90	-	-	42.71–115.97	[5, 58, 59]
* Nasutitermes* spp. (unspecified)	Adult	0.10–18.4	0.07	0.08	0.96–24.6	[2, 8]
* Odontotermes* spp.	Adult	9.2–12.9	6.6–7.6	271.4–515	8.8–13.9	[35]
Termite soldiers/workers	Adult	1.43–17.65	-	-	5.45–32.50	[1, 5]
Termite alates	Adult	1.09	-	-	1.78–3.07	[5]
Termites (mg/kg) (unspecified)	Adult	159.3	77.77	10.433	16.366–205.30	[33, 35, 60]
Silkworms						
* Bombyx mori*	Pupae	16.8–23.00	0.15–2.2	0.71–1.9	3.54–26.00	[2, 8, 18]
* Bombyx mori*	Larvae	17.75	2.08	2.49	9.54	[30]
* Anaphe venata*	Larva	10.00	1.00	40.00	10.00	[8]
* Antheraea pernyi (ug/g)*	Pupae	35.7	7.27		40.0	[61]
* Samia ricinii*	Pupae	7.13	1.78	2.58	23.7	[30]
Silkworm (unspecified)	Pupae	8.80–224.0	1.08–15	7.85–18.0	2.60–326.0	[44, 46, 62]
Stinkbugs						
* Encosternum delegorguei*	Adult	59	28	2	35	[22]
* Euschistus egglestoni*	Adult	59.00	-	-	57.00	[8]
Worms						
* Tenebrio molitor*	Adult	12.99–144	1.68–21	0.64–22	3.69	[13, 31]
* Tenebrio molitor*	Larvae	5.20–49.5	0.61–8.3	0.5–3.2	2.06–20.7	[8, 11, 13, 28, 57]
* Hermetia illucens*	Larvae	4.34–5.62	0.34–0.40	1.13–6.18	6.66	[2, 27, 63]
* Imbrasia* spp.	Larvae	10.83–14.00	0.19–1.40	3.24–5.18	8.74–31.00	[3, 5, 8]
* Usta terpsichore*	Larvae	25.30	2.60	6.70	39.10	[8]

## Insects Supplementation for Trace Elements Deficiency in Food

The deficiency of trace minerals has been observed to result in higher death rates globally over the past two decades [2]. Zinc and iron deficiencies have been observed to pose a serious health concern in many rural communities in developing countries worldwide, especially in Africa [64]. Southern African countries such as South Africa and Zimbabwe are experiencing cases of anaemia due to iron deficiency, especially which occurs mostly in children and pregnant women [22]. According to Motadi et al. [65], many diets consumed by Southern African communities are mainly rich in carbohydrates but deficient in microminerals including zinc and iron. The deficiency in trace elements such as Fe and Zn has been recognised and identified as one of the most problematic conditions that adversely affect the proper functioning of the gastrointestinal tract, central nervous system, and the immune, skeletal, and reproductive systems [25]. This result in anaemia, poor pregnancy outcomes, increased risk of morbidity and mortality, stunted growth in children, and human learning ability [4]. Additionally, the lack of zinc reduces intake and undesirable absorption of food which also results in diarrhoea, pneumonia, and malaria due to abnormal functioning of the immune system [2]. Therefore, there is a need to control and prevent the effect of malnutrition due to deficiency in trace elements through supplementation of human diets with food sources that are rich in minerals [2]. The supplementation of edible insects such as termites is recommended to provide a balanced diet that will improve the health status of people, especially young children and pregnant women in rural communities [5].

Edible insects are recognised as one of the most important food sources for human nutrition worldwide [5]. Insects have been reported to contain large quantities of mineral contents, more than required for human consumption [1]. Their rich micromineral contents can help in addressing mineral deficiencies in human diets [22]. These micronutrients vary depending on the type of insect species [18]. Although many insects contain various minerals, they are mainly rich in iron and zinc [1]. Insect species such as mopane worm and grasshopper *Locusta migratoria* contain a high amount of iron in mg per 100 g of dry matter [47]. The caterpillars are also reported to have high Fe and Zn, which are important nutrients for combating iron deficiency [22]. The high iron contents found in insects could aid in addressing anaemia problems in humans caused by iron deficiency in the body [18, 19, 25]. It has also been reported that dietary supplementation of edible insects could help reduce iron and zinc deficiency faced many marginalised households around the world [30]. It has been reported that the indigenous edible insects such as termites, worms, bugs, locusts, and grasshoppers found in Southern Africa could potentially help alleviate disease caused by a deficiency in trace elements [22].

## Insects Supplementation for Trace Elements Deficiency in Feeds

Feeder insects are considered a good alternative protein source for controversial protein feed [30]. Insects can be utilised successfully due to their high nutritive value. Thus, they are rich in protein, fat, carbohydrate, vitamins, and minerals [18]. According to Orkusz [3], insect species such as *Acheta domesticus*, *Gryllus bimaculatus*, and *Gonimbrasia belina* larvae can be successfully included in animal diets to be utilised as a mineral source in foods that are deficient in various trace elements such as Fe, Zn, Cu, and Mn. Since edible insects are high in micromineral contents, their consumption as part of human diets must be greatly encouraged and promoted [22, 44]. There is a worldwide increase in the consumption of insects, which encourages the potential inclusion in human and animal diets [42].

Insect species including bugs, cockroaches, and crickets have a sufficient amount of trace elements such as Cu, Fe, Mn, and Zn required by different animals such as birds and mammals. [44, 46]. The inclusion of mineral-rich insects in broiler chicken diets could help improve their health and productivity while also reducing feed costs [66]. Although insects are known to provide essential trace elements for human and animal diets, it is reported that they also require mineral supplementation in their diets for growth and development when reared on farms on large scale. This could help manipulate their mineral contents for better utilisation [17]. Therefore, insects could potentially help address malnutrition and poverty in rural communities based on their high nutritive value to ensure food security [18].

## Factors Limiting Utilisation of Trace Elements in Insects

Although edible insects have high nutritive value, it is crucial to consider the presence of toxic and harmful antinutritional substances found in various insects. This includes factors such as allergic and toxic substances, which requires serious attention when utilising insects as food or feed source [30]. It has been reported that some antinutritional factors such as phytate, oxalate, and tannins originate from the insect diet that was consumed and remained in the gastrointestinal tract which adversely affects their availability of trace elements since it will form part of the whole insect during nutrient analysis [14]. As much as the nutritional statuses of the selected edible insects, mopane worms (*Imbrasia* spp.), termites (*Macrotermes* spp.), grasshoppers (*Schistocerca gregaria*), and stinkbugs (*Encosternum delegorguei*), are highly documented, there is less information about the digestibility and availability of minerals from various insects for consumption by humans or animals [16, 21, 67].

Some insects also contain high quantities of heavy metal contents such as lead, arsenic, cadmium, and mercury insects considered harmful to humans. In addition, the metal contaminants greatly influence the levels of trace elements such as iron from various insects during processing [21]. However, insects such as silkworm pupae have been observed to contain the required levels of heavy metal contents recommended for animal and human feeding [46]. Other insects such as caterpillars (mopane worm) may contain huge abnormal amounts of microminerals which can be harmful to humans [22]. Minimum amounts are recommended when supplementing insects in human food or animal feeds [44, 46]. Thus, knowledge about the recommended levels of dietary insect supplementation is required for better utilisation of trace elements such as Fe, Zn, and Mn found in insects [22].

## Effect of Processing Methods on Availability of Trace Elements in Insects

Mineral contents of various insects are highly affected by the processing methods. The common processing methods before consumption include broiling, roasting, or frying [14]. Processing methods have been observed to affect the nutritive composition of various insects by altering their micromineral [14]. According to Mwangi et al. [2], the uptake and absorption of Fe and Zn from insects are influenced by factors such as processing, preservation, preparation, and combinations which adversely inhibit their utilisation by humans. The southern African traditional methods such as drying, salting, and roasting have been reported to result in increased contents of copper, iron, and zinc [14, 22]. Furthermore, roasting, boiling, oven-drying, and freeze-drying of grasshoppers have been observed to produce higher trace element contents such as Fe, Zn, Cu, and Mn [58]. However, the boiling preparation method has been reported to leach trace elements such as iron, zinc, and copper in insects [14]. Frying methods have been observed to reduce zinc contents in edible insects such as worms [39].

## Conclusion

Generally, edible insects are considered a readily available source of proteins, and carbohydrates, and are likely to contain sufficient quantities of trace elements such as iron, zinc, copper, or selenium levels recommended by NRC. Commercially raised feeder insects are validated to contain an adequate amount of trace elements and can be fed to poultry, fish, or pets to maximise growth and reproduction at the lowest cost without regard to the nutrient content of the feeder insect. Most of the literature indicates that insects and arachnids contain sufficient minerals and trace elements. With these essential minerals and trace elements, insects could be used as an alternative source of minerals to supplement the nutritional need of an individual. Changing the diet fed to the insect during growth can alter the nutrient content of the feeder insect. Although nutrients can be altered by changing the diet fed to the insect, the previous studies indicated that not all nutrients can be easily changed, especially minerals such as calcium. Apart from their high mineral content, edible insects are considered affordable, accessible, and can be successfully reared on a large scale to be supplemented with other food sources and help address the mineral deficiency in both human and animal diets. Therefore, the inclusion of insects in human diets worldwide, especially in marginalised communities, can effectively help reduce and prevent many diseases caused by a lack of specified minerals in the body. Further research on the optimal inclusion levels of various insects in human and livestock diets is recommended.

## Data Availability

The data used in this review article was acquired from recently published scientific literature from different journals. Databases were accessed using electronic data sources such as Directory of Open Access Journals (DOAJ), Research Gate, Science Direct, and Google Scholar. In addition, the citations included in articles from the databases were used to search for further relevant literature. The keywords “insect meal”, “edible insects”, and “trace elements” were used in the search engines.
